# Comparison of prognosis and outcomes of catheter ablation versus drug therapy in patients with atrial fibrillation and stable coronary artery disease: A prospective propensity‐score matched cohort study

**DOI:** 10.1002/clc.23699

**Published:** 2021-07-28

**Authors:** Yi‐Kai Cui, Jian‐Zeng Dong, Xin Du, Rong Hu, Liu He, Chang‐Qi Jia, Xu Li, Jia‐Hui Wu, Rong‐Hui Yu, De‐Yong Long, Man Ning, Cai‐Hua Sang, Chen‐Xi Jiang, Rong Bai, Song‐Nan Wen, Nian Liu, Song‐Nan Li, Wei Wang, Xue‐Yuan Guo, Xin Zhao, Song Zuo, Xuan Chen, Shu‐Tao Huang, Hao‐Sheng Wu, Ri‐Bo Tang, Chang‐Sheng Ma

**Affiliations:** ^1^ Department of Cardiology, Beijing Anzhen Hospital Capital Medical University, Beijing Institute of Heart Lung and Blood Vessel Diseases Beijing China

**Keywords:** atrial fibrillation, catheter ablation, coronary artery disease

## Abstract

**Background:**

Atrial fibrillation (AF) and stable coronary artery disease (SCAD) frequently coexist.

**Hypothesis:**

To investigate the prognosis of catheter ablation versus drug therapy in patients with AF and SCAD.

**Methods:**

In total, 25 512 patients with AF in the Chinese AF Registry between 2011 and 2019 were screened for SCAD. 815 patients with AF and SCAD underwent catheter ablation therapy were matched with patients by drug therapy in a 1:1 ratio. Primary end point was composite of thromboembolism, coronary events, major bleeding, and all‐cause death. The secondary endpoints were each component of the primary endpoint and AF recurrence.

**Results:**

Over a median follow‐up of 45 ± 23 months, the patients in the catheter ablation group had a higher AF recurrence‐free rate (53.50% vs. 18.41%, *p* < .01). In multivariate analysis, there was no significant difference between the strategy of catheter ablation and drug therapy in primary composite end point (adjusted HR 074, 95%CI 0.54–1.002, *p* = .0519). However, catheter ablation was associated with fewer all‐cause death independently (adjusted HR 0.36, 95%CI 0.22–0.59, *p* < .01). In subgroup analysis, catheter ablation was an independent risk factor for all‐cause death in the high‐stroke risk group (adjusted HR 0.39, 95%CI 0.23–0.64, *p* < .01), not in the low‐medium risk group (adjusted HR 0.17, 95%CI 0.01–2.04, *p* = .17).

**Conclusions:**

In the patients with AF and SCAD, catheter ablation was not independently associated with the primary composite endpoint compared with drug therapy. However, catheter ablation was an independent protective factor of all‐cause death

## 1 INTRODUCTION

As the most common sustained arrhythmia, atrial fibrillation (AF) has a high possibility to occur together with coronary artery disease (CAD), which is another common cardiovascular disease. Over 20 percent of patients with AF suffered from CAD,^1^ and about 19 percent of patients with CAD had AF.^2^ However, the optimal treatment of AF with CAD remains unclear. Most of previous studies usually focused on the antithrombotic protocol for patients with AF and CAD.

In recent years, catheter ablation has become an important choice for AF treatment. EAST‐AFNET 4 trial reported that early rhythm‐control therapy was associated with a lower risk of a composite of death from cardiovascular causes, stroke, or hospitalization than usual care among patients with early AF and cardiovascular conditions.^3^ Which emphasized the advantages of early rhythm‐control therapy in the management of AF. Despite the fact that catheter ablation was effective for treating AF, whether CAD could affect the outcome of AF catheter ablation remained unclear. In a study from the Leipzig Heart Center, it was found that neither the presence nor severity of CAD could affect the recurrence within 12 months after AF ablation.^4^ Dennis^5^ et al. also found that CAD did not increase the recurrence within 12 months after AF ablation. However, some studies showed the opposite conclusion. Hiraya^6^ et al. found that CAD was an independent risk factor for recurrence of catheter ablation of AF after an average of 44‐months of follow‐up. A retrospective study found that the presence of CAD has no impact on AF recurrence after cryoablation.^7^ The aforementioned studies explored the impact of CAD on the success rate of catheter ablation of AF. It was more important to address the issue of the impact of catheter ablation on the prognosis in the patients with AF and CAD. However, there were only a few studies to address this issue. In EAST‐AFNET4 study, early rhythm control did not reduce the hospitalization with acute coronary syndrome.^3^ A small retrospective cohort study showed that catheter ablation could improve the long‐term prognosis of AF patients who underwent percutaneous coronary intervention (PCI).^8^ The small sample size and the exclusion of patients without PCI lead to low reliability and poor generalization. Here, we aimed to examine the effect of catheter ablation versus drug therapy on long‐term prognosis in the patients with AF and stable coronary artery disease (SCAD) in a prospective cohort study.

## 2 METHODS

### 2.1 Study population

All the patients were screened from the Chinese Atrial Fibrillation Registry (CAFR) study between August 2011 and December 2019. CAFR has been described in details previously.^9^ Briefly, CAFR is a prospective registry study with ongoing enrollments and follow‐up involving 19 tertiary and 12 non‐tertiary hospitals in Beijing, China. Eighteen of the 31 centers had the ability to perform AF ablation. Written consents were obtained from all patients when they enrolled in the CAFR, and the ethics committee approved this study. All the data were collected from the medical record system or through telephone interviews.

Patients would be enrolled in this study if meeting all the following inclusion criteria: (1) age ≥ 18 years; (2) diagnosis of AF; (3) suffered from SCAD. AF was diagnosed by 12‐lead electrocardiogram or 24 hours‐Holter with a record lasting ≥30 seconds. SCAD was defined as a clinical condition with at least one of the following inclusion criteria: myocardial infarction (MI) ≥3 months ago; coronary artery bypass grafting (CABG) or PCI ≥3 months; stable chest pain with proven myocardial ischemia; or previous coronary angiography showing ≥1 coronary stenosis >50% and not require revascularization.^10^

**FIGURE 1:**
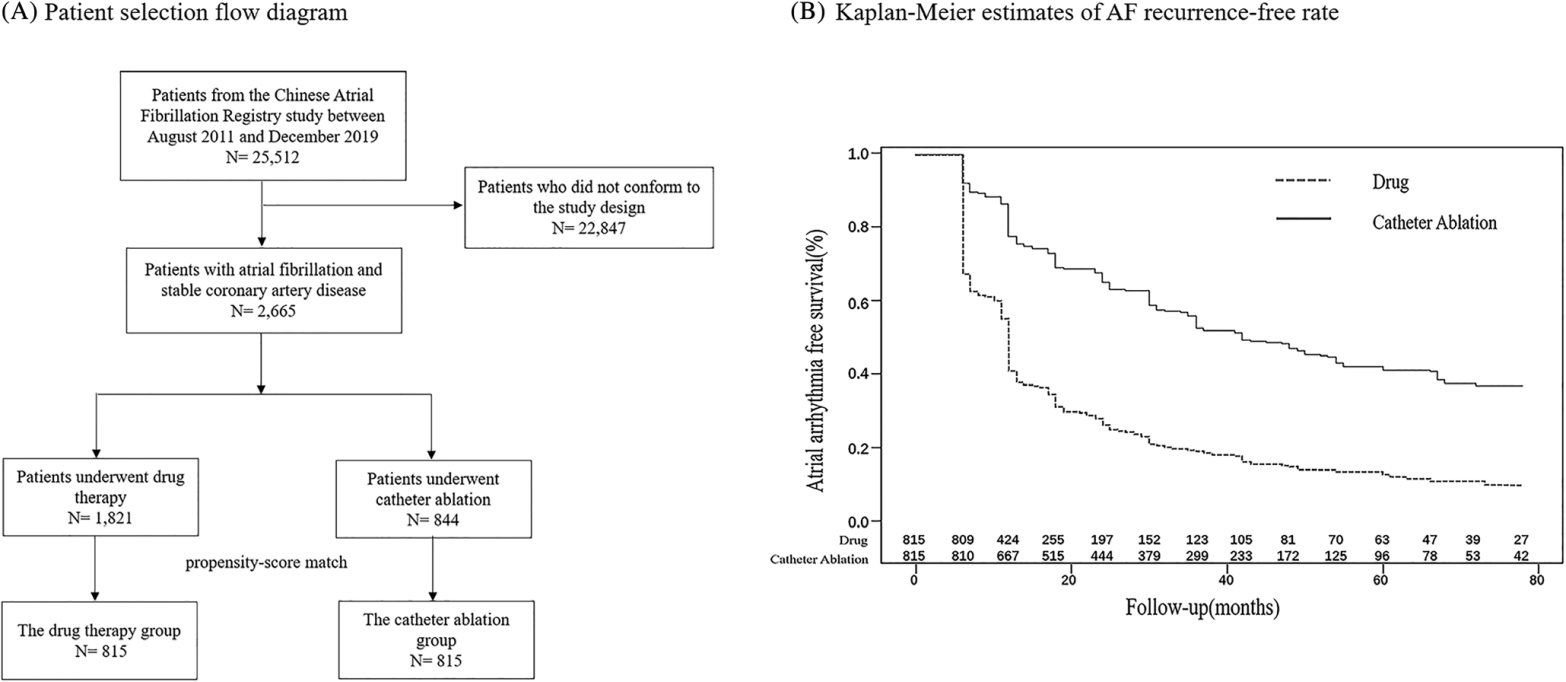
Patient selection flow diagram and Kaplan–Meier estimates of AF recurrence‐free rate. (A) Patient selection flow diagram. (B) Kaplan–Meier survival curve showing atrial arrhythmia recurrence‐free survival after a single ablation. The AF recurrence‐free rate in the catheter ablation group was higher than in the drug therapy group (53.50 vs. 18.41%; *p* < .01). AF, atrial fibrillation

Patients should be excluded if meeting any of the exclusion criteria as the followings: (1) valvular AF; (2) a history of catheter ablation or surgery for AF. In the CAFR, there were 12 104 patients underwent catheter ablation and 13 408 patients underwent drug therapy. Totally, 2665 patients were selected according to the inclusion criteria and exclusion criteria, including 1921 patients with catheter ablation and 844 patients with drug therapy. After propensity‐score matching, 815 pairs of patients in each group were enrolled in the study. The patient selection flow diagram was shown in Figure 1A.

### 2.2 Interventions

In the Catheter Ablation Group, all antiarrhythmic drugs (AAD) except amiodarone were stopped for at least five half‐lives before catheter ablation. The procedure was performed by experienced physicians. AF ablation strategy of our study has been described previously.^11^ The procedures were performed in patients under conscious sedation. A continuous irrigated radiofrequency ablation was performed along each pulmonary vein antrum in order to encircle the ipsilateral pulmonary veins. Procedural end‐points were electrical isolation of all pulmonary veins in patients with paroxysmal AF. In patients with persistent AF, LA roofline, mitral isthmus, and cavotricuspid isthmus were routinely targeted. Pulmonary vein isolation and linear block were identified in sinus rhythm.

Patients underwent catheter ablation were treated with anticoagulant drugs (warfarin, non‐vitamin K antagonist oral anticoagulant) and AADs (amiodarone, sotalol, propafenone) for 3 months after the procedure. AADs were withdrawn in the patients without recurrence 3 months after the catheter ablation. The patients with high risk for stroke were encouraged to continue taking anticoagulation. Antithrombotic strategy was determined by the discussion between physicians and the patients according to the patients' thromboembolism and bleeding risk and the patients' intention. The long‐term antithrombotic and AAD therapy in the ablation group were adopted the data after 3‐month follow‐up.

In the drug therapy group, the long‐term plan of drug therapy was determined by the professional physicians at the first visit. According to the patients' thromboembolism and bleeding risk, antiplatelet drugs (aspirin, clopidogrel, and ticagrelor) or/and anticoagulant drugs (warfarin, non‐vitamin K antagonist oral anticoagulant) would be prescribed by the physicians. According to the patients' symptom and rhythm, the physicians would prescribe AADs (amiodarone, sotalol, propafenone) or/and rate control drugs (beta‐blockers, non‐dihydropyridine calcium channel blockers, digoxin).

### 2.3 Follow‐up

Scheduled follow‐up was implemented at 3, 6, and 12 months after the initiation and every 6 months thereafter. Three strategies were applied to monitor heart rhythm: (i) regular reexamination: 24 hours‐Holter was performed monthly in the first 3 months, which was followed by an ECG and/or 24 hours‐Holter every 6 months thenceforth; (ii) symptom triggered reexamination: patients would record ECGs whenever experienced AF symptoms; and (iii) opportunistic screenings: ECGs recorded for routine examinations or other diseases were involved.

### 2.4 Study endpoints

The primary endpoint was the composite of thromboembolism, coronary events, major bleeding, and all‐cause death. The thromboembolism included ischemic stroke (IS), transient ischemic attack (TIA), and systemic embolism (SE). The coronary events included MI and coronary revascularization. The major bleeding was defined according to the International Society on Thrombosis and Hemostasis (ISTH).^12^ The secondary endpoints included thromboembolism, coronary events, major bleeding, all‐cause death, and AF recurrence. AF recurrence was defined as AF, atrial flutter, or atrial tachyarrhythmia lasting ≥30 seconds record by 12‐lead electrocardiogram or 24 hours‐Holter after a 3‐month blanking period from the initiation in both groups. Once a patient underwent catheter ablation during the follow‐up period, the follow‐up data after the procedure would not be considered in the survival analysis.

### 2.5 Statistical analysis

Propensity‐score matching was used to reduce the selection bias. To generate the propensity score, we made a multiple regression model enrolling 12 baseline variables, including the age, gender, type of AF, MI, heart failure, hypertension, diabetes, history of bleeding, history of thromboembolism, hyperlipidemia, renal insufficiency, liver insufficiency, and chronic obstructive pulmonary disorder. Patients from the catheter ablation group were matched with patients from the drug therapy group by one‐to‐one greedy nearest neighbor matching with a caliper of 0.2.

Continuous variables were presented as means ± SD or medians and quartiles. Categorical variables were presented as numbers and proportions. Continuous variables in normal distribution were compared with Student's *t*‐test or Wilcoxon test if not in normal distribution. The Chi‐square tests were used to compare categorical variables. Kaplan–Meier was used to calculate AF recurrence‐free survival. The incidence rates of outcome events were calculated by dividing the numbers of events by person‐years. Cox regression analysis was used to find the independent predictors of primary endpoint. The multivariate analysis model included variables with a *p*‐value < .05 in univariate analysis, including ablation, age, type of AF, diabetes, heart failure, history of bleeding, history of thromboembolism, estimated glomerular filtration rate (EGFR), left atrial diameter, left ventricular end‐diastolic diameter (LVEDD), left ventricular ejection fraction (LVEF), digoxin, statins, antithrombotic drugs. A *p*‐value < .05 was considered statistically significant. All statistical analyses were performed using SAS software, version 9.4 (SAS Institute, Cary, NC).

## 3 RESULTS

### 3.1 Baseline characteristics

Totally, 815 pairs of patients with AF and SCAD were enrolled with a mean duration of 45 ± 23 months follow‐up. The baseline characteristics of the patients were shown in Table 1. Most of the variables were comparable between the two groups for the reason of propensity score matched. Compared with the drug therapy group, the patients in the catheter ablation group were younger, had longer history of AF, higher LVEF, lower prevalence of smoke and drink. There was significant difference of antithrombotic drugs between the two groups.

**TABLE 1:** Baseline characteristics of the two groups

Baseline characteristics	Catheter ablation group (n = 815)	Drug therapy group (n = 815)	*p* value
Age, years, mean ± SD	66.1 ± 8.2	67.0 ± 8.9	.04
Female, n (%)	248 (30.4)	263 (32.3)	.42
Body mass index, kg/m^2^, mean ± SD	26.0 ± 3.7	25.7 ± 3.4	.11
Smoker, n (%)	125 (15.3)	157 (19.3)	.04
Alcohol consumption, n (%)	110 (13.5)	151 (18.5)	<.01
Paroxysmal AF, n (%)	575 (70.6)	573 (70.3)	.96
AF duration, year, median (IQR)	2.4 (0.6–5.3)	1.7 (0.1–5.3)	<.01
Previous coronary events			
MI, n (%)	145 (17.8)	151 (18.5)	.70
PCI, n (%)	425 (52.2)	387 (47.5)	.06
CABG, n (%)	63 (7.7)	73 (9.0)	.37
CHA_2_DS_2_‐VASc score, median (IQR)	3.0 (2.0–4.0)	3.0 (2.0–4.0)	.07
Hypertension, n (%)	565 (69.3)	600 (73.6)	.06
Diabetes, n (%)	284 (34.9)	274 (33.6)	.60
Heart failure, n (%)	71 (8.7)	70 (8.6)	.93
Previous bleeding, n (%)	38 (4.7)	37 (4.5)	.91
Previous thromboembolic events, n (%)	123 (15.1)	129 (15.8)	.68
LDL‐C, mmol/L, median (IQR)	2.2 (1.7–2.7)	2.2 (1.8–2.8)	.23
ALT, U/L, median (IQR)	22.0 (16.0–29.0)	21.0 (15.0–30.0)	.20
Cr, μmol/L, median (IQR)	79.3 (68.1–91.0)	79.5 (68.7–91.0)	.99
eGFR, ml/min/1.73 m^2^	103.3 (89.2–119.7)	101.6 (86.0–122.2)	.59
COPD, n (%)	8 (1.0)	9 (1.1)	.81
Left atrial diameter (mm), median (IQR)	40.0 (37.0–44.0)	41.0 (37.0–45.0)	.12
LVEDD (mm), median (IQR)	48.0 (45.0–51.0)	48.0 (45.0–52.0)	.08
LVEF, (%) (IQR)	64.0 (60.0–68.0)	62.0 (57.0–68.0)	<.01
Beta‐blockers, n (%)	363 (44.5)	453 (55.6)	<.01
Amiodarone, n (%)	60 (7.4)	56 (6.9)	.70
Propafenone, n (%)	31 (3.8)	28 (3.4)	.70
Sotalol, n (%)	17 (2.1)	17 (2.1)	1.00
Non‐DHP CCBs, n (%)	31 (3.8)	57 (7.0)	<.01
Digoxin, n (%)	7 (0.9)	27 (3.3)	<.01
ACEI/ARB, n (%)	302 (37.1)	319 (39.1)	.39
Statin, n (%)	499 (61.2)	507 (62.2)	.68
Antithrombotic drugs			<.01
Antiplatelet agent, n (%)	361 (44.3)	249 (30.6)	
Anticoagulant, n (%)	165 (20.3)	215 (26.4)	
Antiplatelet agent + anticoagulant, n (%)	10 (1.2)	18 (2.2)	

Abbreviations: AF, atrial fibrillation; ALT, alanine aminotransferase; CABG, coronary artery bypass grafting; CHA2DS2‐VASc, congestive heart failure, hypertension, age 75 years (doubled), diabetes, stroke/transient ischemic attack/thromboembolism (doubled), vascular disease (prior myocardial infarction, peripheral artery disease, or aortic plaque), age 65–75 years, sex category (female); COPD, chronic obstructive pulmonary diseases; eGFR, estimated glomerular filtration rate; LDL‐C, low‐density lipoprotein cholesterol; LVEDD, left ventricular end‐diastolic diameter; LVEF, left ventricular ejection fraction; MI, myocardial infarction; non‐DHP CCBs, non‐dihydropyridine calcium channel blockers; PCI, percutaneous coronary intervention.

### 3.2 General characteristics of catheter ablation

All the patients in the catheter ablation group achieved pulmonary vein isolation. 118 (21%) of the 575 patients with paroxysmal AF and 208 (87%) of the 240 patients with non‐paroxysmal AF underwent additional linear ablation, fractionated potentials ablation or superior vena cava isolation. Complications occurred in 13 patients (1.60%), including six pericardial tamponade, 1 pericardial effusion, 1 acute heart failure, 2 stroke, 1 puncture hematoma, 1 great saphenous venous thrombosis, and 1 femoral arteriovenous fistula.

With a mean duration of 54.5 ± 24.0 months follow‐up, after a single ablation, the AF recurrence‐free rate was higher in the catheter ablation group than the drug therapy group (53.50 vs. 18.41%; *p* < .01; Figure 1B).

### 3.3 Study endpoints

Clinical adverse events were shown in Table 2. After an average of 45 ± 23 months of follow‐up, in univariate analysis, the composite primary endpoint (3.28 per 100 person‐years vs. 5.27 per 100 person‐years; *p* < .01) and all‐cause mortality (0.88 per 100 person‐years vs. 2.76 per 100 person‐years; *p* < .01) were significantly lower in the catheter ablation group. There were no significant differences of other secondary endpoints including thromboembolism, coronary events, and major bleeding between the two groups. With regard to the component of thromboembolism, 62 patients in the drug therapy group experienced thromboembolism events (46 IS, 8 TIA, and 8 SE) and 60 patients in the catheter ablation group experienced thromboembolism events (39 IS, 13 TIA, and 8 SE). There was no significant difference in the type of thromboembolism events between the two groups (*p* = .42).

**TABLE 2:** Clinical outcomes during follow‐up

	Catheter ablation group		Drug therapy group			
	n	IR (95%CI)	n	IR (95%CI)	HR (95%CI)	*p* value^a^
Primary end point	89	3.28 (2.66–4.04)	176	5.27 (4.54–6.11)	0.66 (0.51–0.86)	<.01
Secondary end point						
Thromboembolism	60	2.19 (1.70–2.82)	62	1.80 (1.41–2‐31)	1.26 (0.88–1.80)	.21
Major bleeding	13	0.46 (0.27–0.79)	30	0.85 (0.59–1.22)	0.58 (0.30–1.12)	.11
Coronary events	4	0.14 (0.05–0.37)	14	0.39 (0.23–0.66)	0.55 (0.18–1.68)	.30
All‐cause death	25	0.88 (0.59–1.30)	98	2.76 (2.27–3.37)	0.33 (0.22–0.52)	<.01

Abbreviation: IR, incidence rates per 100 person‐years; n, number of events.

^a^ *p* value for HR.

The univariate and multivariate analyses of risk factors for primary endpoint were shown in Table 3. The univariate analysis revealed that catheter ablation, age, type of AF, diabetes mellitus, heart failure, previous bleeding, previous thromboembolism, EGFR, left atrial diameter, LVEDD, LVEF, digoxin, statins, anticoagulants were risk factors for the primary endpoint. The multivariate analysis showed that age (adjusted HR, 1.04 [1.02–1.06]; *p* < .01), diabetes (adjusted HR, 1.50 [1.13–2.02]; *p* < .01), previous bleeding (adjusted HR, 1.91 [1.15–3.19]; *p* = .01), previous embolism (adjusted HR, 1.76 [1.25–2.46]; *p* < .01), eGFR (adjusted HR, 0.99 [0.988–0.999]; *p* = .02), LVEF (adjusted HR, 0.98[0.96–0.996]; *p* = .02), and digoxin (adjusted HR, 2.07[1.13–3.80]; *p* = .02) were independently associated with the primary endpoint. In multivariate analysis, catheter ablation therapy was not an independent risk factor for the primary endpoint (adjusted HR, 0.74[0.54–1.002]; *p* = .0519). Catheter ablation therapy was an independent protective factor for all‐cause death (adjusted HR, 0.36 [0.22–0.59]; *p* < .01) in the multivariate model.

**TABLE 3:** Univariate and multivariate analysis for the primary endpoint

	Univariate		Multivariate	
	HR (95%CI)	*p* value	HR (95%CI)	*p* value
Age	1.05 (1.03–1.06)	<.01	1.04 (1.02–1.06)	<.01
Female	1.06 (0.82–1.36)	.68		
Obesity (BMI ≥ 28 kg/m^2^)	0.85 (0.63–1.15)	.30		
Smoker	0.86 (0.62–1.19)	.86		
Alcohol consumption	0.74 (0.52–1.04)	.09		
Paroxysmal AF	0.75 (0.58–0.97)	.03	0.93 (0.66–1.30)	.66
AF duration	1.01 (0.99–1.03)	.66		
MI	1.19 (0.88–1.61)	.26		
PCI	1.20 (0.94–1.52)	.15		
CABG	1.45 (0.99–2.13)	.054		
Hypertension	1.04 (0.79–1.38)	.78		
Diabetes	1.34 (1.05–1.72)	.02	1.50 (1.12–2.02)	<.01
Heart failure	2.08 (1.50–2.88)	<.01	1.21 (0.79–1.86)	.37
Previous bleeding	1.65 (1.02–2.67)	.04	1.91 (1.15–3.18)	.01
Previous thromboembolic events	1.72 (1.29–2.28)	<.01	1.76 (1.25–2.46)	<.01
Peripheral arterial disease	0.49 (0.07–3.48)	.48		
LDL‐C	0.99 (0.84–1.17)	.93		
ALT	1.002 (0.996–1.007)	.56		
eGFR	0.99 (0.985 0.995)	<.01	0.993 (0.988–0.999)	.02
COPD	1.04 (0.26–4.20)	.95		
Left atrial diameter	1.04 (1.02–1.06)	<.01	1.01 (0.99–1.04)	.37
LVEDD	1.03 (1.01–1.05)	.02	1.01 (0.98–1.04)	.46
LVEF	0.96 (0.95–0.98)	<.01	0.98 (0.959–0.996)	.02
Beta‐blockers	0.97 (0.76–1.24)	.80		
Amiodarone	0.93 (0.56–1.54)	.78		
Propafenone	0.70 (0.33–1.49)	.36		
Sotalol	0.21 (0.03–1.51)	.12		
Non‐DHP CCBs	1.23 (0.79–1.92)	.36		
Digoxin	2.92 (1.78–4.77)	<.01	2.07 (1.13–3.81)	.02
ACEI/ARB	0.91 (0.71–1.17)	.47		
Statin	0.74 (0.58–0.95)	.02	0.66 (0.49–0.90)	<.01
Antiplatelet agent + anticoagulant vs. non‐antithrombotic	2.59 (1.26–5.32)	<.01	2.35 (0.97–5.70)	.06
Antiplatelet agent vs. non‐antithrombotic	0.84 (0.63–1.13)	.84	0.92 (0.65–1.32)	.66
Anticoagulant vs. non‐antithrombotic	1.31 (0.97–1.76)	.07	1.21 (0.83–1.77)	.31
Catheter ablation	0.66 (0.51–0.86)	<.01	0.74 (0.543–1.002)	.0519
AF recurrence	1.21 (0.91–1.60)	.19		

Abbreviations: AF, atrial fibrillation; ALT, alanine aminotransferase; CABG, coronary artery bypass grafting; COPD, chronic obstructive pulmonary diseases; eGFR, estimated glomerular filtration rate; LDL‐C, low‐density lipoprotein cholesterol; non‐DHP CCBs, non‐dihydropyridine calcium channel blockers; LVEDD, left ventricular end‐diastolic diameter; LVEF, left ventricular ejection fraction; MI, Myocardial infarction; PCI, percutaneous coronary intervention.

### 3.4 Subgroup analysis

The patients were divided into two different stroke risk subgroups based on the CHA2DS2‐VASc score. The patients with CHA2DS2‐VASc score ≤ 1 in men or ≤ 2 in women were grouped into the low‐medium risk group, and the patients with CHA2DS2‐VASc score ≥ 2 in men or ≥ 3 in women were grouped into the high‐risk group. As it was shown in Table 4, catheter ablation was not a risk factor for the primary endpoint in either group. For all‐cause death, catheter ablation was an independent risk factor in the high‐risk group (adjusted HR, 0.39 [0.23–0.64]; *p* < .01). However, catheter ablation was not an independent risk factor for all‐cause death in the low‐medium risk group (adjusted HR, 0.17 [0.01–2.04]; *p* = .16).

**TABLE 4:** Primary outcomes and all‐cause death in the subgroup analysis stratified by stroke risk

	Events, No. (%)					
	Catheter ablation group	Drug therapy group	Unadjusted HR (95%CI)	*p* value	Adjusted HR^a^ (95%CI)	*p* value
Primary outcomes						
Low‐medium risk group^b^	6 (4.3)	12 (10.5)	0.49 (0.18–1.30)	.15	0.68 (0.21–2.20)	.52
High‐risk group^b^	83 (12.3)	164 (23.4)	0.7 (0.54–0.91)	<.01	0.77 (0.56–1.05)	.10
All‐cause death						
Low‐medium risk group^b^	1 (0.7)	5 (4.4)	0.17 (0.02–1.48)	.17	0.17 (0.01–2.04)	.16
High‐risk group^b^	24 (3.6)	93 (13.3)	0.36 (0.23–0.56)	<.01	0.39 (0.23–0.64)	<.01

^a^ Adjusted for ablation, age, type of AF, diabetes, heart failure, history of bleeding, history of embolism, estimated glomerular filtration rate, left atrial diameter, left ventricular end‐diastolic diameter, left ventricular ejection fraction, digoxin, statins, antithrombotic drugs.

^b^ The patients with CHA2DS2‐VASc score ≤ 1 in men or ≤ 2 in women were grouped into the low‐medium risk group, and the patients with CHA2DS2‐VASc score ≥ 2 in men or ≥ 3 in women were grouped into the high‐risk group.

## 4 DISCUSSION

### 4.1 Major findings

To the best of our knowledge, this study was the largest study that exploring catheter ablation therapy versus drug therapy on the outcomes in the patients with AF and SCAD based on the real‐world data. The main findings of this study are: (1) Compared with drugs therapy, catheter ablation was more effective at maintaining sinus rhythm for the patients with AF and SCAD. (2) In multivariate analysis, catheter ablation was not independently associated with fewer primary composite endpoints of thromboembolism, coronary events, major bleeding, or all‐cause death. (3) For a secondary endpoint, after adjustment for the confounders in multivariate analysis, catheter ablation was an independent protective factor of all‐cause death after adjusting for other potential risk factors.

### 4.2 AF and CAD

AF and CAD were closely related, and they shared some same risk factors, such as hypertension, diabetes, sleep apnea syndrome, obesity, smoking, and inflammation. CAD could affect the blood supply of atria, and coronary artery revascularization could reduce the recurrence of AF.^13^ Alasady^14^ et al. found that CAD affecting the atrial artery was independently associated with AF. By measuring coronary artery blood with a microcatheter, it was found that left atrial hypoperfusion existed in patients with lone AF.^15^ The atrial ischemia caused by CAD could lead to ion channel disorder, electrical and mechanical reconstruction, fibrosis and scarring, and even local conduction block that could induce and maintain AF.^16^ On the other hand, the incidence rate of CAD was higher in patients with AF than heathy persons. AF could predict CAD, independent of conventional risk factors.^17^ Besides, AF could predict MI in patients with and without CAD.^18^, ^19^ AF impaired myocardial perfusion, which could be improved by cardioversion.^20^ With controlled ventricular rate, AF still could independently reduce atrial hypoperfusion by its irregularity.^21^ Furthermore, AF could damage the endothelial cell by its special hemodynamics, and these findings were reversible after electrical cardioversion.^20^, ^22^ Endothelial dysfunction was probably attributed to the rise of the asymmetric dimethylarginine levels and the downregulation of endothelial nitric oxide synthase expression, which were caused by AF.^23^, ^24^, ^25^ AF could also lead to increase in platelet activation and the level of plasma inflammatory mediators, which were risk factors for coronary events.^25^ These pathological states for CAD could also be reversed by maintaining sinus rhythm.^23^, ^25^

### 4.3 Catheter ablation for AF

In the recently published CABANA study, catheter ablation was not superior to drug therapy on the primary endpoint, which was a composite of death, disabling stroke, serious bleeding, and cardiac arrest.^26^ In the CABANA study, only about 19% of participants suffered from CAD, the outcomes of patients with CAD and AF have not been analyzed in the CABANA study. The CASTLE‐AF study, a randomized controlled trial, confirmed that catheter ablation was superior to drug therapy in patients with concurrent AF and heart failure. However, the information about impacts of CAD on the prognosis was also limited.^27^ Accordingly, the data of catheter ablation on the prognosis in the patients with concurrent AF and SCAD were very limited. This study added some information about the issue of on the outcomes in the patients with AF and SCAD.

A small observational trial of catheter ablation therapy vs. medical therapy in the patients with AF and prior coronary intervention provided evidence that catheter ablation may reduce adverse events, including acute coronary syndrome requiring hospitalization, stroke, pulmonary embolism and mortality.^8^ Our study found that catheter ablation could not improve the composite primary endpoints in the patients with AF and SCAD. However, catheter ablation could reduce all‐cause mortality. The difference between the two studies might be due to the different study population. The exclusion of patients without PCI in the aforementioned study led to poor generalization. Our study with larger sample size and longer follow‐up may influence the treatment choice for the patients with AF and SCAD. Furthermore, our study showed that whether catheter ablation therapy was associated with fewer all‐cause mortality depended on the stroke risk based on CHA2DS2‐VASc scores.

### 4.4 Drug therapy

According to recent guidelines, patients with AF and CAD were suggested to take an anticoagulant or at last an antiplatelet drug.^28^ In our study, 37.5% of patients did not take any antithrombotic drugs, which did not comply with the guidelines. In accordance with this study, a retrospective study from China which included 21 450 patients with acute coronary syndrome, only 70.03% of the patients started antiplatelet therapy in the first 30 days. And among the patients with antiplatelet drugs, 85.0% of them stopped the drugs after an average of 117.4 days.^29^ In our study, only 21.5% of patients in catheter ablation group and 28.6% of patients in drug therapy group took oral anticoagulation therapy, which was not in accordance with the guidelines.^30^ Previous study showed that only 36.5, 28.5, and 21.4% of patients in the CAFR with CHA2DS2‐VASc scores ≥2, 1, and 0 underwent oral anticoagulation therapy.^31^ In other Chinese AF centers, according to studies conducted between 2017 and 2020, the proportions of oral anticoagulation use were from 13.9 to 35.6%.^32^, ^33^, ^34^, ^35^ As a current status, oral anticoagulation was significantly underused in patients in China. In addition, the application of antiplatelet drugs or anticoagulation in Chinese patients was hard to sustain.^31^, ^36^ The problems with patients' compliance, the misgiving about the risk of bleeding, and the high cost of the non–vitamin K antagonist oral anticoagulants were possible reasons for the lack of anticoagulation therapy. This study revealed the gap between the clinical practice in the real world and the guidelines in China, which might contribute to the high incidence of adverse events in our study.

### 4.5 Coronary events

The incidence of coronary events was relatively low in this study. In a similar study by Chong et al., after catheter ablation of AF, 2.3% of patients who had undergone PCI experienced MI, and 15.8% of patients experienced unstable angina.^8^ In the AFIRE study focusing on the patients with AF and SCAD, 0.9% of patients experienced MI, and 1.4% of patients experienced revascularization for unstable angina after a mean 24.1‐month follow‐up.^37^ Compared with these studies, the incidence of coronary events in our study was lower. The possible reasons were as the following. (1) The baseline characteristics of the patients were different in these studies. 18.2% of the patients in this study had previous MI. In Chong's study, 33.9% of the patients had previous non‐ST‐segment elevation MI. In the AFIRE study, 33.9% of patients had previous MI. In addition, the mean age of the patients in the AFIRE study was 74 years old, which was higher than that of our study. (2) MI was a significant cause of sudden cardiac death. In this study, many patients died without determining the causes of death, which might underestimate the incidence of coronary events.

### 4.6 Thromboembolism events and major bleeding events

In most registries on AF, stroke was much less common than bleeding. However, the incidence of thromboembolism events was higher than that of major bleeding in this study. Compared with bleeding, Chinese were more likely to suffer from thromboembolism events. It was reported in an observational study enrolling 9806 AF patients with anticoagulation therapy, the incidence of IS and SE was 6.5%, and the incidence of intracranial hemorrhage and gastrointestinal bleeding was 4.9%.^38^ In another retrospective study, the incidence of IS and SE was 2.8%, and the incidence of major bleeding events was 1.1% in the patients with AF treated with dabigatran in China.^39^ These studies from China provided the evidences that there might be racial difference in the incidence of adverse events. In this study, about 37.5% of patients did not take any antithrombotic drugs, which might cause a further increase in thromboembolism events and reduce major bleeding events.

### 4.7 Limitations

Some main limitations existed in this study. (1) Our research was a prospective cohort study rather than a randomized controlled trial. Selection bias could exist for the inherent deficiency of observational study. For balancing patients' characteristics, propensity‐score matching was used to reduce the bias. There were also some slight differences of the baseline characteristics between the two groups. Multivariate analysis was used to adjust for potential confounding factors. (2) A gap of drugs therapy between the clinical practice in the real world and the guidelines appeared in this study. However, the application of drugs in our study was in accordance with other Chinese studies. This study emphasized the effects of catheter ablation versus drug therapy on the prognosis in the patients with AF and SCAD in the real world. (3) The time span of our study's enrollment was long, from 2011 to 2019. The standard of drug treatment and the catheter ablation technology has partly changed. However, the enrollment and follow‐up were consistent and ongoing in the CAFR.

## 5 CONCLUSION

In conclusion, in the patients with AF and SCAD, compared with drug therapy, after adjusted the cofounders, catheter ablation was not significantly associated with fewer primary composite endpoints of thromboembolism, coronary events, major bleeding, and all‐cause death. However, catheter ablation could lead to fewer all‐cause death.

## Data Availability

Research data are not shared.

## References

[1] Nieuwlaat R , Capucci A , Camm AJ , et al. Atrial fibrillation management: a prospective survey in esc member countries: the euro heart survey on atrial fibrillation. Eur Heart J. 2005;26(22):2422‐2434. 16204266 10.1093/eurheartj/ehi505

[2] Zielonka A , Tkaczyszyn M , Mende M , et al. Atrial fibrillation in outpatients with stable coronary artery disease: results from the multicenter recent study. Pol Arch Med Wewn. 2015;125(3):162‐171. 25644126 10.20452/pamw.2709

[3] Kirchhof P , Camm AJ , Goette A , et al. Early rhythm‐control therapy in patients with atrial fibrillation. N Engl J Med. 2020;383(14):1305‐1316. 32865375 10.1056/NEJMoa2019422

[4] Kornej J , Hindricks G , Arya A , et al. Presence and extent of coronary artery disease as predictor for af recurrences after catheter ablation: the Leipzig heart center af ablation registry. Int J Cardiol. 2015;181:188‐192. 25528310 10.1016/j.ijcard.2014.12.039

[5] den Uijl DW , Boogers MJ , Compier M , et al. Impact of coronary atherosclerosis on the efficacy of radiofrequency catheter ablation for atrial fibrillation. Eur Heart J Cardiovasc Imaging. 2013;14(3):247‐252. 22815375 10.1093/ehjci/jes144

[6] Hiraya D , Sato A , Hoshi T , et al. Impact of coronary artery disease and revascularization on recurrence of atrial fibrillation after catheter ablation: importance of ischemia in managing atrial fibrillation. J Cardiovasc Electrophysiol. 2019;30(9):1491‐1498. 31190437 10.1111/jce.14029

[7] Liu L , Zhao D , Zhang J , et al. Impact of stable coronary artery disease on the efficacy of cryoballoon ablation for the atrial fibrillation. Am J Med Sci. 2019;358(3):204‐211. 31307767 10.1016/j.amjms.2019.06.004

[8] Chong E , Chang HY , Chen YY , et al. When atrial fibrillation co‐exists with coronary artery disease in patients with prior coronary intervention ‐ does ablation benefit? Heart Lung Circ. 2016;25(6):538‐550. 26839165 10.1016/j.hlc.2015.12.001

[9] Du X , Ma C , Wu J , et al. Rationale and design of the chinese atrial fibrillation registry study. BMC Cardiovasc Disord. 2016;16:130. 27266985 10.1186/s12872-016-0308-1 PMC4897860

[10] Sorbets E , Steg PG , Young R , et al. Β‐blockers, calcium antagonists, and mortality in stable coronary artery disease: an international cohort study. Eur Heart J. 2019;40(18):1399‐1407. 30590529 10.1093/eurheartj/ehy811 PMC6503455

[11] Dong JZ , Sang CH , Yu RH , et al. Prospective randomized comparison between a fixed '2c3l' approach vs. stepwise approach for catheter ablation of persistent atrial fibrillation. Europace. 2015;17(12):1798‐1806. 25957039 10.1093/europace/euv067

[12] Schulman S , Kearon C . Definition of major bleeding in clinical investigations of antihemostatic medicinal products in non‐surgical patients. J Thromb Haemost. 2005;3(4):692‐694. 15842354 10.1111/j.1538-7836.2005.01204.x

[13] Murakami N , Tanno M , Kokubu N , et al. Distinct risk factors of atrial fibrillation in patients with and without coronary artery disease: a cross‐sectional analysis of the boreas‐cag registry data. Open Heart. 2017;4(1):e000573. 28123767 10.1136/openhrt-2016-000573 PMC5255559

[14] Alasady M , Abhayaratna WP , Leong DP , et al. Coronary artery disease affecting the atrial branches is an independent determinant of atrial fibrillation after myocardial infarction. Heart Rhythm. 2011;8(7):955‐960. 21338715 10.1016/j.hrthm.2011.02.016

[15] Skalidis EI , Hamilos MI , Karalis IK , Chlouverakis G , Kochiadakis GE , Vardas PE . Isolated atrial microvascular dysfunction in patients with lone recurrent atrial fibrillation. J Am Coll Cardiol. 2008;51(21):2053‐2057. 18498961 10.1016/j.jacc.2008.01.055

[16] Sinno H , Derakhchan K , Libersan D , Merhi Y , Leung TK , Nattel S . Atrial ischemia promotes atrial fibrillation in dogs. Circulation. 2003;107(14):1930‐1936. 12668526 10.1161/01.CIR.0000058743.15215.03

[17] Weijs B , Pisters R , Haest RJ , et al. Patients originally diagnosed with idiopathic atrial fibrillation more often suffer from insidious coronary artery disease compared to healthy sinus rhythm controls. Heart Rhythm. 2012;9(12):1923‐1929. 22885921 10.1016/j.hrthm.2012.08.013

[18] Bayturan O , Puri R , Tuzcu EM , et al. Atrial fibrillation, progression of coronary atherosclerosis and myocardial infarction. Eur J Prev Cardiol. 2017;24(4):373‐381. 27837151 10.1177/2047487316679265

[19] Ruddox V , Sandven I , Munkhaugen J , Skattebu J , Edvardsen T , Otterstad JE . Atrial fibrillation and the risk for myocardial infarction, all‐cause mortality and heart failure: a systematic review and meta‐analysis. Eur J Prev Cardiol. 2017;24(14):1555‐1566. 28617620 10.1177/2047487317715769 PMC5598874

[20] Range FT , Schäfers M , Acil T , et al. Impaired myocardial perfusion and perfusion reserve associated with increased coronary resistance in persistent idiopathic atrial fibrillation. Eur Heart J. 2007;28(18):2223‐2230. 17604290 10.1093/eurheartj/ehm246

[21] Kochiadakis GE , Skalidis EI , Kalebubas MD , et al. Effect of acute atrial fibrillation on phasic coronary blood flow pattern and flow reserve in humans. Eur Heart J. 2002;23(9):734‐741. 11978000 10.1053/euhj.2001.2894

[22] Yoshino S , Yoshikawa A , Hamasaki S , et al. Atrial fibrillation‐induced endothelial dysfunction improves after restoration of sinus rhythm. Int J Cardiol. 2013;168(2):1280‐1285. 23269316 10.1016/j.ijcard.2012.12.006

[23] Lim HS , Willoughby SR , Schultz C , et al. Successful catheter ablation decreases platelet activation and improves endothelial function in patients with atrial fibrillation. Heart Rhythm. 2014;11(11):1912‐1918. 25068571 10.1016/j.hrthm.2014.07.030

[24] Cai H , Li Z , Goette A , et al. Downregulation of endocardial nitric oxide synthase expression and nitric oxide production in atrial fibrillation: potential mechanisms for atrial thrombosis and stroke. Circulation. 2002;106(22):2854‐2858. 12451014 10.1161/01.cir.0000039327.11661.16

[25] Lim HS , Willoughby SR , Schultz C , et al. Effect of atrial fibrillation on atrial thrombogenesis in humans: impact of rate and rhythm. J Am Coll Cardiol. 2013;61(8):852‐860. 23333141 10.1016/j.jacc.2012.11.046

[26] Packer DL , Mark DB , Robb RA , et al. Effect of catheter ablation vs antiarrhythmic drug therapy on mortality, stroke, bleeding, and cardiac arrest among patients with atrial fibrillation: the cabana randomized clinical trial. JAMA. 2019;321(13):1261‐1274. 30874766 10.1001/jama.2019.0693 PMC6450284

[27] Marrouche NF , Brachmann J , Andresen D , et al. Catheter ablation for atrial fibrillation with heart failure. N Engl J Med. 2018;378(5):417‐427. 29385358 10.1056/NEJMoa1707855

[28] Knuuti J , Wijns W , Saraste A , et al. 2019 ESC guidelines for the diagnosis and management of chronic coronary syndromes. Eur Heart J. 2020;41(3):407‐477. 31504439 10.1093/eurheartj/ehz425

[29] Liu X , He X , Wu J , Luo D . Initiation and persistence with antiplatelet agents among the patients with acute coronary syndromes: a retrospective, observational database study in China. Patient Prefer Adherence. 2019;13:2159‐2169. 31908423 10.2147/PPA.S228065 PMC6925556

[30] Hindricks G , Potpara T , Dagres N , et al. 2020 ESC guidelines for the diagnosis and management of atrial fibrillation developed in collaboration with the european association for cardio‐thoracic surgery (EACTS). Eur Heart J. 2021;42(5):373‐498. 32860505 10.1093/eurheartj/ehaa612

[31] Chang SS , Dong JZ , Ma CS , et al. Current status and time trends of oral anticoagulation use among chinese patients with nonvalvular atrial fibrillation: the chinese atrial fibrillation registry study. Stroke. 2016;47(7):1803‐1810. 27283198 10.1161/STROKEAHA.116.012988

[32] Xiang X , Cao Y , Sun K , et al. Real world adherence to oral anticoagulant in non‐valvular atrial fibrillation patients in China. Curr Med Res Opin. 2018;34(2):255‐261. 29022745 10.1080/03007995.2017.1391760

[33] Liu T , Yang HL , Gu L , et al. Current status and factors influencing oral anticoagulant therapy among patients with non‐valvular atrial fibrillation in Jiangsu province, China: a multi‐center, cross‐sectional study. BMC Cardiovasc Disord. 2020;20(1):22. 31948390 10.1186/s12872-020-01330-6 PMC6964080

[34] Yu R , Xi H , Lu J , et al. Real‐world investigation on discontinuation of oral anticoagulation after paroxysmal atrial fibrillation catheter ablation in China. Ann Palliat Med. 2020;9(3):940‐946. 32434352 10.21037/apm-20-565

[35] Cheng X , Zhou X , Song S , et al. Ethnicity and anticoagulation management of hospitalized patients with atrial fibrillation in Northwest China. Sci Rep. 2017;7:45884. 28393880 10.1038/srep45884 PMC5385550

[36] Wang Y , Nichol MB , Yan BP , et al. Descriptive analysis of real‐world medication use pattern of statins and antiplatelet agents among patients with acute coronary syndrome in Hong Kong and the USA. BMJ Open. 2019;9(7):e024937. 10.1136/bmjopen-2018-024937 PMC6661883 31315855

[37] Yasuda S , Kaikita K , Akao M , et al. Antithrombotic therapy for atrial fibrillation with stable coronary disease. N Engl J Med. 2019;381(12):1103‐1113. 31475793 10.1056/NEJMoa1904143

[38] Law SWY , Lau WCY , Wong ICK , et al. Sex‐based differences in outcomes of oral anticoagulation in patients with atrial fibrillation. J Am Coll Cardiol. 2018;72(3):271‐282. 30012320 10.1016/j.jacc.2018.04.066

[39] Rong G , Huang Y , Wang L , Liang H , Wang H . Persistence, effectiveness and safety of dabigatran in "real‐world" chinese patients with nonvalvular atrial fibrillation. Heart Vessels. 2020;35(7):977‐984. 32006091 10.1007/s00380-020-01565-5

